# Acupuncture for gastrointestinal urticaria: A protocol for systematic review and network meta-analysis

**DOI:** 10.3389/fmed.2022.998655

**Published:** 2022-10-14

**Authors:** Yiwei Li, Leixiao Zhang, Qiong Wang, Yanli Deng

**Affiliations:** ^1^Sichuan Second Chinese Medicine Hospital, Chengdu, China; ^2^Department of Integrated Traditional Chinese and Western Medicine, Sichuan University West China Hospital, Chengdu, China; ^3^Department of Pain Treatment, Suining Central Hospital, Suining, China

**Keywords:** gastrointestinal urticaria, acupuncture, protocol, network meta-analysis, systematic review

## Abstract

**Introduction:**

The purpose of this review is to evaluate the effectiveness and safety of acupuncture in the treatment of patients with gastrointestinal urticaria (GU) and to provide a clinician's guide to GU treatment options.

**Methods and analysis:**

We plan to search multiple databases (i.e., PubMed, EMBASE, Springer, Web of Science, Cochrane Library, China National Knowledge Infrastructure, Chinese Biomedical Literature Database and Wanfang Database) for studies published before September 1, 2022. We will electronically search for all relevant studies concerning clinical acupuncture treatments of GU, including unpublished conference articles and other gray literature. The language limit of this systematic review is Chinese and English. Any reports of clinical randomized controlled trials of acupuncture for the treatment of GU will be included in the study. Two researchers will perform independent data extraction to increase the quality of the data extraction. The primary outcome was the Urticaria Activity Score 7 (UAS7). Abdominal visual analog scale (VAS) for abdominal pain, dermatological life quality index (DLQI), the total effective rate, recurrence rate, and occurrence of adverse events were secondary outcomes. We will use RevMan V.5.3 statistical software for pairwise meta-analysis and ADDIS V.1.16.8 software for Bayesian network meta-analysis. If feasible, meta-regression and subgroup analyses will also be performed to address the potential causes of inconsistency and heterogeneity. We will conduct a GRADE assessment of the quality of evidence for the interventions included in this review.

**Discussion:**

This study may validate acupuncture as an alternative therapy for the effective treatment of GU.

**Trial registration number:**

PROSPERO CRD42022333977.

## Introduction

Urticaria is a disease characterized by the development of wheals, with intense pruritus, and with or without angioedema (1). It is a clinically refractory and relapsing disease that is common in dermatology. According to the time of onset (6 weeks as the boundary) into acute and chronic urticaria. Gastrointestinal urticaria (GU) is a special type of chronic urticaria characterized by chronic diarrhea, weight loss, abdominal distension, and anorexia (2, 3). Previous studies have shown that chronic urticaria is often related to various inflammatory or infectious diseases, including gastrointestinal infections, such as Helicobacter pylori infection or nasopharyngeal bacterial infections (4, 5). Bile reflux, atrophic gastritis, and other gastrointestinal diseases, as well as other associated diseases, such as those of the hepatobiliary duct, esophagus, stomach, and duodenum, are also related to urticaria (6). The overall prevalence of GU in patients ranges from 26.2 to 50% in adults (7, 8) and is ~12.8% in children (9). Therefore, gastrointestinal inflammation causes urticaria, a finding which has been confirmed by previous studies. In a recent clinical investigation involving patients with chronic spontaneous urticaria (CSU), more than a quarter of the patients with CSU had gastrointestinal symptoms that may be related to a greater disease burden and higher serum pancreatin levels (7). In one study of 155 patients, 47.6% reported headache/fatigue and 26.2% reported gastrointestinal complaints; 41.7% of emergency department visits in the past year, 65% of patients were absent for more than 1 day and 84.5% of patients used oral corticosteroids (7). Since urticaria is primarily an outpatient condition, the cost of medication alone can be as high as $1,280 (10). Daytime fatigue, low mood, decreased study or work productivity, and high medical costs resulting from hives severely damage these patients. Since GU has multiple etiologies and hazards, the diagnosis and treatment of GU remains an important clinical challenge in the future.

The digestive system is an important “immune organ.” It is known as the largest “bacteria and antigen library” and a gathering place of many lymphatics and immune factors (11). Several immune cells, such as T/B lymphocytes, macrophages, and mast cells, can be produced in the human gastrointestinal mucosa. Immune cells produced in the intestine stimulate the differentiation of immune cells and the emergence of immune mediators, which play an important role in regulating the mucus layer and lymphatic structure of the intestine (12). Internal disturbances of external factors can cause a continuous imbalance of the intestinal flora, thereby inducing metabolic disorders of the body's system (13, 14). Genome-wide transcriptome analysis of the gut revealed upregulation of several genes associated with mast cells and eosinophils, an observation confirmed by demonstrating increased mast cell numbers for IgE (+) and FCRε (+) in the colon and skin (15). High-fat diet-induced obesity enhances food-induced allergic responses associated with dysregulated gut effector mast Cell responses, increased gut flooding, and gut dysbiosis (16). Studies have found that wind mass-like changes in patients with chronic urticaria are mainly concentrated in the gastric mucosa, and these changes interact, appearing or disappearing together (17). Hypothalamic dysfunction can decrease the gastric motility and secretory function of the gastric mucosa and induce abdominal pain and diarrhea; however, these symptoms can be reduced through continuous improvement in the gastrointestinal function (18).

The three major barriers of the gastrointestinal tract are important fortresses of the body. For instance, the consumption of alcohol and the abuse of antibiotics and preservatives can disrupt the balance of the intestinal flora, indirectly leading to immune dysfunction (19). Therefore, defects in intestinal barrier function can trigger an abnormal immune response to the destruction of various internal and external factors, which may evoke the onset of chronic urticaria. There are many T cells in the tissues of the skin and gastrointestinal tract, and abnormally expressed cytokines are often observed in patients (20, 21). Although most IL-17 (Th17) and IFγ (Th1) T cells exist in the gastrointestinal tract (22), the abnormal expression of cytokines was shown to be closely related to the severity of GU (23). Researchers found that patients with *H. pylori* infection had a greater urticaria severity and more episodes (24). Recently, a systematic review and meta-analysis including 22 studies with 1,385 patients with CSU showed that the effectiveness of *H. pylori* eradication therapy in suppressing CSU symptoms significantly (25) reduced the recurrence rate after a three-month treatment (26).

Due to the numerous causes of the disease, it is difficult to determine a precise treatment for this disease. Therefore, major improvement in clinical signs and symptoms remains the focus. Although second-generation antihistamines, hormones, and immunosuppressants are commonly used and are relatively effective (1), their associated problems, such as multiple side effects, high cost, and high recurrence rates, often plague patients (27). Therefore, to improve clinical treatment methods, it is necessary to find a complementary or alternative therapy with obvious curative effects and no side effects.

For 2,500 years, acupuncture has been used to treat GU (28, 29). In 2010, acupuncture and moxibustion were officially included in the “List of Representatives of Intangible Cultural Heritage of Humanity,” and it has become one of the complementary and alternative treatments recognized worldwide. In acupuncture, needles are inserted into acupoints to regulate the blood of the meridians throughout the body to achieve the balance of yin and yang. With the deepening understanding of modern medicine, the mechanism underlying the acupuncture treatment of GU has been consistently validated. Specifically, acupuncture can regulate body fluid and cellular immunity, enhance the phagocytic ability of macrophages and B cells, correct the balance of Th1 and Th2 lymphocytes, reduce mast cell degranulation (30), and reduce vascular permeability (31). In addition, acupuncture treatment can also activate endogenous morphine-like substances and inhibit and block the transmission of pain and itching signals to achieve analgesia and alleviate itching. Furthermore, it can help maintain intestinal flora and immune homeostasis (32) and promote the release of beneficial bacteria that reduce the content of pathogenic bacteria and enhance the stability of the flora itself, thereby exerting a benign protective effect on the diversity and abundance of the intestinal flora (33). Through these effects, acupuncture treatment can effectively treat gastrointestinal symptoms (34).

Although research on pruritus diseases has only gradually emerged, several reports based on the results of many randomized controlled trials (RCTs) on the treatment of GU (35–37) have been published. Yet, we found through our previous literature review that, to date, there has been no network meta-analysis of acupuncture for the treatment of GU. Therefore, our purpose for conducting this review is to provide clinicians with evidence-based and safe alternative treatment methods for GU.

## Methods and analysis

Under the provisions of the Preferred Reporting Items for Systematic Reviews and Meta-Analyses (PRISMA) Protocol statement guidelines, we have constructed the protocol report and implementation review (38, 39). Details about acupuncture and control interventions will be extracted from Standards for Reporting Interventions in Clinical Trials of Acupuncture.

### Study registration

The proposed systematic review was registered on PROSPERO (CRD42022333977).

### Data sources

Under the provisions of the Cochrane Handbook Guidelines, we have formulated the search strategy for this systematic review (40). Specifically, we will search various databases electronically for all clinical acupuncture treatments of GU Research before September 1, 2022. These databases included PubMed, EMBASE, Springer, Web of Science, Cochrane Library, China National Knowledge Infrastructure, Chinese Biomedical Literature Database and Wanfang Database. We will then search for gray literature, including unpublished conference articles. The language limit of this systematic review is Chinese and English. Any studies concerning clinical randomized controlled trials of acupuncture for the treatment of GU will be included in the study. The following search terms will be used: urticaria, chronic urticaria, gastrointestinal urticaria, diarrhea, weight loss, bloating, abdominal pain, anorexia, acupuncture, hand needles, electroacupuncture, skin needles, and ear needles (Supplementary Table 1).

We will also retrieve and include unpublished data related to this review, such as meeting minutes and unpublished articles. To search for unpublished but completed clinical trials, we will log on to the WHO International Clinical Trials Registry Platform (ICTRP) (http://www.who.int/ictrp/en/), National Institutes of Health Clinical Registry (https://www.who.int/ictrp/en/; https://www.clinicaltrials.gov/) and China Clinical Registry (http://www.chictr.org/en/). We will contact investigators by email in an attempt to obtain the latest clinical data from these randomized controlled trials.

### Eligibility criteria

#### Population

We will use the EAACI/GA^2^LEN/EDF/WAO guidelines for the definition, classification, diagnosis, and management of urticaria to define patients with GU with gastrointestinal symptoms (1). No restrictions will be made regarding sex, age, race, education, and economic status.

#### Interventions

There are many acupuncture methods in Traditional Chinese Medicine. Our review will include studies that utilize hand needles, electroacupuncture, skin needles, and ear needles. In addition, the so-called placebo needle treatment, which includes placebo needles, non-acupoints, and placebo acupuncture at non-acupoints, among others. We will exclude studies using the following GU treatment methods: acupressure, moxibustion, laser acupuncture, dry acupuncture, or transcutaneous electrical nerve stimulation. We will complete the following comparisons:

i. Acupuncture will be compared with waiting for treatment.
ii. Acupuncture will be compared with placebo acupuncture.
iii. Acupuncture will be compared with other active therapies.

In each RCT included in our systematic review, we will evaluate the educational background and clinical experience of the relevant acupuncturist, treatment processes, treatment time, treatment frequency and follow-up (41).

### Outcome measures

#### Primary outcome

The primary outcome of this systematic review is the urticaria activity score 7 (UAS7) (1). UAS7 is an effective tool and “gold standard” for monitoring urticaria activity, which has been recommended many times by EAACI/GA^2^LEN/EDF/WAO and international guidelines jointly initiated by representatives of 42 countries. UAS7 was determined by the number of wind masses and pruritus degree group, and the higher the score, the more frequent the urticaria activity (42).

#### Secondary outcomes

The secondary outcomes will include a visual analog scale (VAS) score for abdominal pain (43), dermatology life quality index (DLQI) (44), total effective rate {Clinical criteria for judging the efficacy of acupuncture treatment will be set at four levels: (1) Clinically cured, (2) Markedly effective, (3) Effective, and (4) Invalid. The total effective rate will be calculated as: Total effective rate (%) = [(number of patients clinically cured + markedly effective + effective)/number of patients] × 100% (45, 46)},recurrence rate, and occurrence of adverse events.

### Study design

RCTs of acupuncture treatment for GU will be included in our study. Additionally, we will exclude other studies, including non-randomized controlled trials, quasi-randomized trials, animal studies, case reports, and self-control.

### Data extraction

**Figure 1:**
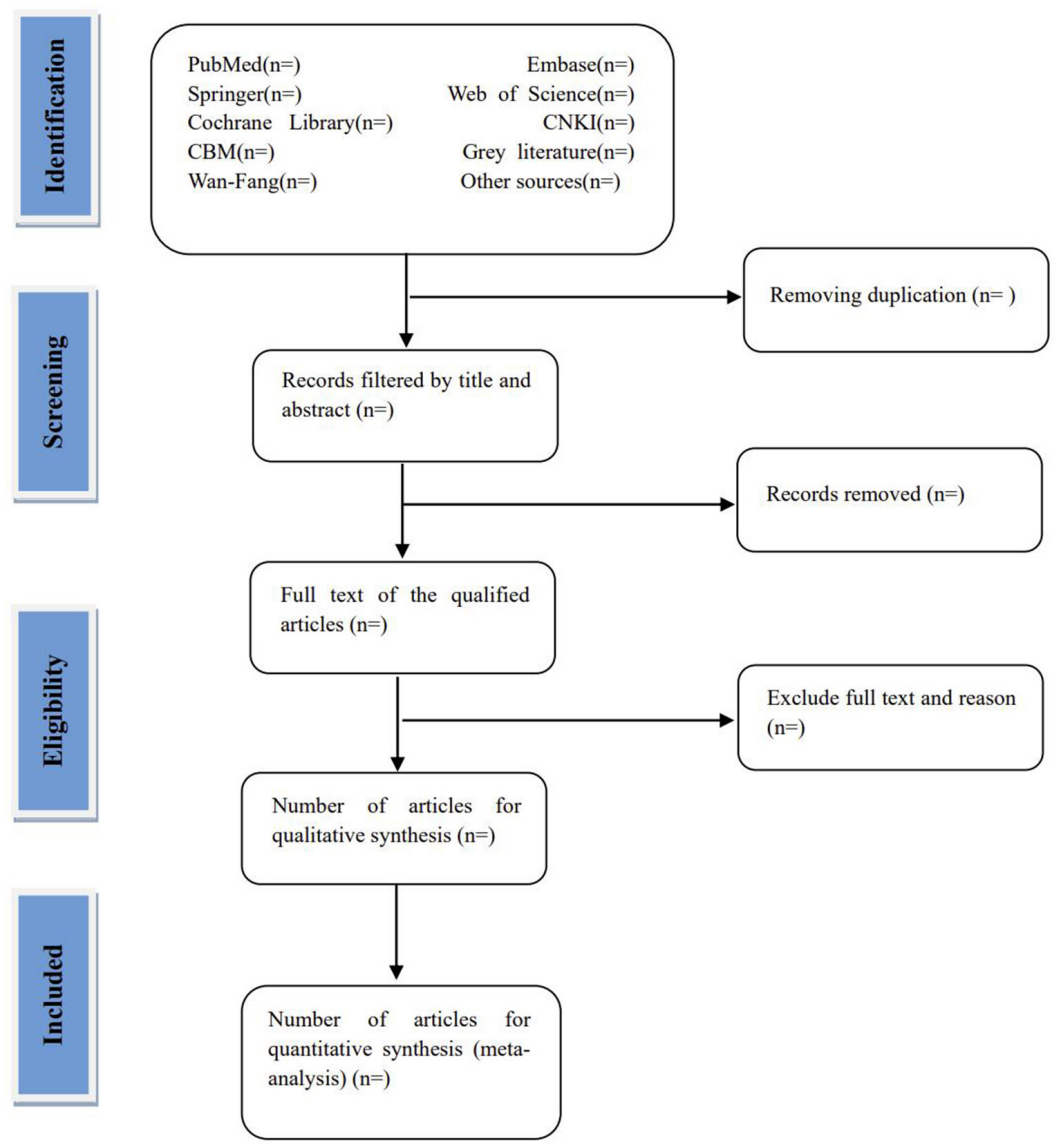
Flow diagram of studies identified.

Regardless of whether the RCT is from an electronic or manual search, we will add it into the database created in EndNote software (V.X8, Clarivate, Philadelphia, Pennsylvania, USA). The test topics, abstracts, and keywords will be added to the database by two independent reviewers (YL and QW). The two reviewers will then search for any studies from this database that meet the inclusion criteria of this review. For studies that meet the inclusion criteria, the reviewers will read the full texts for a comprehensive evaluation. The trials that are excluded will be individually explained and marked. During the review process, a third author (YD) and two reviewers (YL and QW) will discuss and resolve any discrepancies between the two reviewers. If the dispute remains unresolved, the author of the original text will be contacted *via* email for complete clarification. The review process is shown in the PRISMA flowchart (Figure 1).

Each reviewer will complete the evaluation independently. They will extract the data of the articles that they select and fill in a data sheet. The datasheet will mainly include general information, participants, methods, intervention measures, results, adverse events, conflicts of interest, ethics, and other study information. The datasheet will provide a basis for completing the systematic analysis. In addition, when some data is missing, we will contact the author *via* email for information. When there is disagreement, the two examiners will discuss and resolve such issues. When such a disagreement cannot be rationally resolved, an arbitration agreement will be conducted by the third examiner (YD).

Regarding the data in the three-arm test, if it conforms to our experimental design, two groups will be compared directly, one group will be reused, and a sensitivity analysis will be done later (A and B are combined, and then compared with C to form a 2: 1 pairwise comparison). If only two of the three groups meet the purpose of our research, then directly extract the two groups, and finally declare the reasons for extraction and non-extraction.

### Quality assessment

The quality of included studies was judged as low to moderate using the Cochrane's risk of bias (RoB) tool V.5.1.0 (YL and LZ). Risks of bias will be assessed according to the following domains: sequence generation, allocation concealment, blinding, result indicators, and other deviations, among others. Our review will use a three-level risk assessment. A low-level deviation is represented by L (low), uncertainty level is represented by U (unclear), and a high-level deviation is represented by H (high). If the results of the two independent reviewers' risk assessments are inconsistent, an arbitration agreement will be conducted by the third examiner (YD). All risk assessment results will be presented in the form of a table.

### Missing data

To obtain missing data, we will contact the first or corresponding author of the trials *via* email. To determine whether the evaluation results are inconsistent, we will conduct intent analysis and sensitivity analysis on the data of all participants.

### Assessment of similarity and consistency

The similarity will be addressed by comparing variables in the study. Variables will include patient characteristics (age and severity of disease), trial design (risk of blindness and bias), and intervention (type, session, intensity, and duration).

Assumptions of consistency will be assessed using local and global methodologies. The node splitting method will be used to evaluate and calculate the *p*-value (47). *P* > 0.05 will be considered statistically insignificant (48). In addition, the design-by-treatment interaction model and chi-square test will be used to assess the inconsistencies across the entire network.

### Pairwise meta-analysis

Software RevMan (V.5.3, The Nordic Cochrane Center for The Cochrane Collaboration, Copenhagen, Denmark) will be the primary tool used to conduct the pairwise meta-analysis of this systematic review. This instrument will measure the treatment effect of 95% confidence intervals (CIs). The effect measures will include the risk ratio for dichotomous data and the mean difference for continuous data. The heterogeneity within each pairwise comparison will be assessed with the Q test and the I^2^ value. The premise of using a fixed-effects model to merge data is that the *p* > 0.10, and the I^2^ value is <50%. If this premise is not met, we will then use the random-effects model.

### Network meta-analysis

We will use ADDIS (V.1.16.8, Drugis, Groningen, NL) software for a network meta-analysis (49). A Bayesian network meta-analysis will be performed using random-effects models and Markov chain Monte Carlo methods to integrate direct and indirect evidence. The parameters will be set to four chains for emulation, and each chain will have 50,000 simulation iterations. The model convergence will be evaluated by visual examination of trace plots and consideration of the Gelman–Rubin statistic. STATA (V.15.0, StataCorp LLC; College Station, TX, USA) software will be used to generate network diagrams and compare each result (48). For each specific outcome, we will rank the effects of different acupuncture methods to determine which has the most effective surface and mean rank under the cumulative ranking curve and its 95% CI.

### Assessment of heterogeneity

The evaluation of treatment-effect heterogeneity will be completed in accordance with the requirements of the Cochrane Intervention System Evaluation Manual. We will visually check the forest map and use the heterogeneity χ^2^ test and Higgins' I^2^ statistic to evaluate the heterogeneity of this review. When heterogeneity occurs, a meta-analysis with random-effects models can be used to estimate the overall treatment effect. Subgroup analysis and meta-regression analysis can help us find the causes of any heterogeneity between research results in the meta-analysis.

### Meta-regression, subgroup analysis, and sensitivity analysis

Meta-regression will be performed using the following covariables: (1) initial severity of the disease; (2) sample size; (3) average age; and (4) duration, frequency, and course of acupuncture. Because many methods have been applied for acupuncture treatment of GU, we will conduct a subgroup analysis based on the heterogeneity, study type, and clinical differences of acupuncture types (such as hand needles, ear needles, skin needle,or electroacupuncture). Multiple sensitivity analyses will be performed to assess the robustness of the results and to evaluate whether any particular study contributes most of the heterogeneity. If the heterogeneity remains too large after data pooling or subgroup analysis and the interpretation is unclear, we will consider not performing a meta-analysis and only complete a systematic review.

### Grading the quality of evidence

We will conduct a GRADE assessment of the quality of evidence for the interventions included in this review. The main evaluation factors of this review include bias, consistency, directness, accuracy, publication bias, and attachment risk. Evidence evaluation of all the results will be divided into four levels: high, medium, low, or very low.

### Assessment of publication bias

If more than 10 studies are included, we will use STATA (V.15.0) software to perform Begg's and Egger's tests to assess the asymmetry of the funnel chart. The funnel chart will be used to detect publication bias. The meta-analysis will be considered to have statistically significant publication bias if the *p* < 0.05.

## Discussion

As a special type of chronic urticaria, GU deserves due attention because of its complex clinical symptoms. The efficacy of acupuncture has been widely confirmed in China and abroad. An increasing number of researchers, especially acupuncturists, are focusing on the treatment of GU. Initial findings from previous studies have confirmed the effectiveness and safety of acupuncture and moxibustion for the treatment of GU (35–37). Acupuncture is a kind of physical stimulation, which has the characteristics of multi-channel and multi-target action. The biomechanical stimulation signals of acupuncture can be transformed into bioelectrical and chemical signals. Interferes with various cells and nerve fibers in the skin and muscles; Changes in signaling pathways and transcriptional activities of cells, mediators and receptors; and lead to inhibition of peripheral and central itch transmission (50). Secondly, acupuncture can help maintain intestinal flora and immune homeostasis, enhance the stability of the flora itself, and protect the diversity and abundance of intestinal flora (32, 33). However, there is still a lack of objective and convincing supporting evidence. Although searching Chinese and English databases may generate language sampling bias, we plan to assess the literature systematically and critically to engender a complete review of the literature. The network meta-analysis will compare the effectiveness of all available acupuncture therapies and rank the probability of acupuncture methods relative to each domain of GU. The significance of this study is that it can provide a basis for clinicians to choose acupuncture as an alternative therapy for the effective treatment of GU to achieve favorable clinical results.

## Strengths and limitations

- This is the first network meta-analysis to evaluate the effectiveness and safety of acupuncture treatment for GU. The results of the review can provide a useful resource to clinicians seeking alternative treatment options for GU.
- This review will help advance the scientific research on the effectiveness of acupuncture treatment for pruritus associated with urticaria diseases.
- Because the treatment methods of acupuncture are diverse and varied, different acupuncture treatment methods and different standards of efficacy evaluation may lead to significant heterogeneity.

## Data availability statement

The original contributions presented in the study are included in the article/Supplementary Materials, further inquiries can be directed to the corresponding author.

## Ethics statement

As the data included in our systematic review contain no personalized information, no ethical approval will be required for the review. The protocol for our systematic review will be published in journals after the publication review process.

## Author contributions

YD is the guarantor. YL developed the protocol methodology. QW and LZ were responsible for planning the statistical analysis. The original manuscript was jointly completed by YL, LZ, and QW and modified by YD. All authors contributed to the article and approved the submitted version.

## Funding

This study was funded by the National Key Research and Development Program of the China-Key Project Research on Modernization of Traditional Chinese Medicine–International Cooperation Research on Evaluation of Acupuncture Advantage Disease (Nos. 2017YFC1703600 and 2017YFC1703605), Scientific and Technical Research Project of Sichuan Administration of Traditional Chinese Medicine: Study on the mechanism of Yishen Xiaojie Prescription delaying nephrotic syndrome renal fibrosis (No. 2020JC111), the hospital-level project of the Second Hospital of Traditional Chinese Medicine of Sichuan Province: Clinical efficacy study on the treatment of senile chronic constipation based on the development of constipation powder based on the homology of medicine and food (No. 20-6-595), and the Natural Science Foundation of Sichuan Province-Exploring the multi-dimensional regulatory mechanism of acupuncture on the default network of chronic spontaneous urticaria based on spatiotemporal analysis technology (No. 2022NSFSC1492).

## Conflict of interest

The authors declare that the research was conducted in the absence of any commercial or financial relationships that could be construed as a potential conflict of interest.

## Publisher's note

All claims expressed in this article are solely those of the authors and do not necessarily represent those of their affiliated organizations, or those of the publisher, the editors and the reviewers. Any product that may be evaluated in this article, or claim that may be made by its manufacturer, is not guaranteed or endorsed by the publisher.
