# Supplementary material for: Acupuncture for gastrointestinal urticaria: A protocol for systematic review and network meta-analysis
Source: Front Med (Lausanne). 2022 Oct 14;9:998655. doi: 10.3389/fmed.2022.998655 (PMC9614266; doi:10.3389/fmed.2022.998655)
Supplement: Supplementary file 1 [file Data_Sheet_1.pdf]

Supplementary Table 1

Search strategy used in PubMed	
**No.**	**Search terms**
#1	Acupuncture. Mesh.
#2	Acupuncture therapy. Mesh.
#3	Acupoints.ti,ab
#4	Hand needles.ti,ab
#5	Skin needles.ti,ab
#6	Ear needles.ti,ab
#7	Electroacupuncture.ti,ab
#8	#1 OR #2 - #7
#9	Randomised controlled trial.pt
#10	Controlled clinical trial.pt
#11	Randomised.ti,ab
#12	Randomly.ti,ab
#13	Placebo.ti,ab
#14	Sham.ti,ab
#15	Trial.ti,ab
#16	Groups.ti,ab
#17	#9 OR #10 - #16
#18	Chronic urticaria.Mesh
#19	Hives.ti,ab
#20	Nettle-Rash.ti,ab
#21	Fong-Tzen-Kwai.ti,ab
#22	Wind-Rash-Patch.ti,ab
#23	Gastrointestinal.ti.ab
#24	Weight loss.ti.ab
#25	Diarrhea.ti.ab
#26	Abdominal bloating.ti.ab
#27	Abdominal pain.ti.ab
#28	Anorexia.ti.ab
#29	#18 OR #19 - #28
#30	#71 AND #17 AND #29

Embase

#1 (((('acupuncture'/exp OR acupuncture OR ' hand '/exp OR hand) AND ('acupuncture'/exp OR acupuncture) OR 'electroacupuncture'/exp OR electroacupuncture OR 'acupuncture'/exp OR acupuncture) AND ('therapy'/exp OR therapy) OR ear) AND ('acupuncture'/exp OR acupuncture) OR 'acupuncture'/exp OR acupuncture) AND (' skin '/exp OR skin)

#2 ('pruritis'/exp OR pruritis) AND ('itching'/exp OR itching) AND ('itch'/exp OR itch)

#3 Chronic urticaria

#4 Gastrointestinal

#5(randomised AND controlled AND trial OR controlled)
AND clinical AND trial OR randomised OR randomly OR trial OR groups

#6 #1 AND #2 AND #3 AND #4 AND #5

Springer Link

acupuncture or hand needles or electroacupuncture or ear needles or skin needles and Acupuncture Treatment and Pruritus and Randomized Controlled Trial and Gastrointestinal urticaria

Web of science

#1 TS=(“acupuncture *” OR “hand needles *” OR “electroacupuncture *” OR “skin needles *” OR “auricular acupuncture *” OR “ear needles *”)

#2 TS=((“Pruritis *” OR “Itching *” OR “Itch *”))

#3 TS=((“Chronic urticaria *”))

#4 TS=((((((Randomised controlled trial) OR (Controlled clinical trial)) OR (Randomised)) OR (Randomly)) OR (Trial)) OR (Groups))

#5 TS=((“Gastrointestinal *”))

#6 #5 AND #4 AND #3 AND #2 AND #1

Cochrane library

#1 MeSH descriptor: [Acupuncture] explode all trees

#2 MeSH descriptor: [Acupuncture, Ear] explode all trees

#3 MeSH descriptor: [Electroacupuncture] explode all trees

#4 MeSH descriptor: [Acupuncture Therapy] explode all trees

#5 MeSH descriptor: [Acupuncture Points] explode all trees

#6 #1 OR #2 OR #3 OR #4 OR #5 in Cochrane Reviews, Cochrane Protocols, Trials, Clinical Answers, Editorials, Special Collections

#7 MeSH descriptor: [Chronic urticaria] explode all trees

#8 MeSH descriptor: [Randomized Controlled Trial] explode all trees

#9 MeSH descriptor: [Randomized Controlled Trials as Topic] explode all trees

#10 MeSH descriptor: [Control Groups] explode all trees

#11 #7 OR #8 OR #9 OR #10 in Cochrane Reviews, Cochrane Protocols, Trials, Clinical Answers, Editorials, Special Collections

#12 MeSH descriptor: [Gastrointestinal] explode all trees

#13 #6 and #11 and #12

CNKI

(((SU = '瘙痒'+'痒') OR (TKA = '瘙痒'+'痒')) AND ((SU = '针' + '针灸' + '针刺' + '电针' + '手针' + '手捻针' + '毫针' + '体针' + '耳针'+ '皮肤针') OR (TKA = '针' + '针灸' + '针刺' + '电针' + '手针' + '手捻针' + '毫针' + '体针' + '耳针'+ '皮肤针')) AND ((SU = 'RCT') OR (SU% = '随机' + '对照' + '分配' + '临床' + '试验' + '多中心' + '随访' + 'CCT') OR (TKA = '随机' + '对照' + '分配' + '临床' + '试验' + '多中心' + '随访' + 'RCT' + 'CCT'))) AND ((SU%= '荨麻疹') OR (TKA = '荨麻疹')) AND ((SU%= '胃肠') OR (TKA = '胃肠')) NOT ((SU%= '动物实验') OR (TKA = '鼠') OR (TKA = '兔'))

CBM

((针灸 OR 电针 OR 耳针 OR 皮肤针 OR 针刺) AND (胃肠)) AND (荨麻疹) AND (随机 OR 对照 OR 试验 OR 分组)

Wan-fang

主题=(针灸 OR 针刺 OR 电针 OR 耳针 OR 皮肤针) AND (荨麻疹) AND (胃肠) AND (随机 OR 对照 OR 试验 OR 分组)
